# Synthesis and Characterization of Superconducting Ca_1−_*_x_*Na*_x_*FFeAs

**DOI:** 10.3390/ma7031984

**Published:** 2014-03-07

**Authors:** Klaus K. Wolff, Larysa Shlyk, Markus Bischoff, Eva Rose, Rainer Niewa, Thomas Schleid

**Affiliations:** 1 Institut für Anorganische Chemie, Universität Stuttgart, Pfaffenwaldring 55, 70569 Stuttgart, Germany; E-Mails: wolff@iac.uni-stuttgart.de (K.K.W.); shlyk@iac.uni-stuttgart.de (L.S.); bischoff@iac.uni-stuttgart.de (M.B.); niewa@iac.uni-stuttgart.de (R.N.); 2 1. Physikalisches Institut, Universität Stuttgart, Pfaffenwaldring 57, 70550 Stuttgart, Germany; E-Mail: rose@pi1.physik.uni-stuttgart.de

**Keywords:** high-*T_c_*, iron arsenides, superconductors

## Abstract

A representative of the fluoride-containing iron pnictide high-temperature superconductors, namely CaFFeAs, was doped with sodium up to the composition Ca_0.86_Na_0.14_FFeAs for the first time. Single crystals with an edge length in the range of 0.1 – 2.0 mm were obtained via solid-state and flux syntheses, respectively. The composition of the crystals was verified by means of single crystal X-ray diffractometry and energy dispersive X-ray spectroscopy (EDX). Measurements of the electrical resistivity, as well as the magnetization on a crystal of Ca_0.89_Na_0.11_FFeAs both show a transition to the superconducting state on cooling to 34.5 K. Investigations of the upper critical fields reveal an anisotropy ratio of about five. The lattice parameters and molar volumes increase with rising sodium content. This effect is clearly observable for the *c*-axis and the volume, whereas the increase of the *a*-axis is rather minor.

## 1. Introduction

With the detection of superconductivity in iron pnictides in 2008 [1,2], a new class of high-temperature superconductors [3] was found 22 years after the discovery of this phenomenon in oxocuprates [4]. The so-called 1111 phases can be derived from LaOFeAs [5,6] with a tetragonal ZrSiCuAs-type structure, in which isotypical cationic [LaO]^+^ and anionic [FeAs]^−^ layers alternate. An isotypic class is the series, *Ae*FFeAs (*Ae* = Ca, Sr, Eu) [7–9], which results from the exchange of oxygen by fluorine and lanthanum by divalent alkaline-earth metals or europium, respectively. Meanwhile, several structurally related types of such iron pnictides became known [10]. Analogous to the oxocuprates, the superconductivity occurs in two-dimensional antiferromagnetically ordered layers, based on iron-centered edge-linked pnictogen tetrahedra. Within the 1111 compounds, superconductivity is only observed if a partial reduction (electron doping) or oxidation (hole doping) in the [FeAs]^−^ layers occurs [11]. Critical temperatures between *T_c_* = 26 and 43 K for LaO_1−_*_x_*F*_x_*FeAs [2,12] and 55 to 58 K for SmO_1−_*_x_*F*_x_*FeAs [13,14] were achievable for fluoride-doped samples, whereas the strontium substituted oxide La_1–_*_x_*Sr*_x_*OFeAs [15] exhibits a transition temperature of *T_c_* = 25 K.

Up to now, only electron-doped derivatives of the fluorine containing the 1111 compound CaFFeAs, like CaFFe_1−_*_x_*Co*_x_*As [7] and Ca_1−_*_x_Ln_x_*FFeAs (*Ln* = Pr, Nd) [16], are reported to superconduct. After the discovery of superconductivity in 1111 phases, hole doping on LaOFeAs with Ca^2+^ was already investigated without the detection of any superconducting phases [2]; the reason why electron doping was claimed as a critical factor for the generation of superconductivity in this system at that time. However, this view was disproven by hole-doped La_1−_*_x_*Sr*_x_*OFeAs with *T_c_* = 25 K for *x* = 0.13 three months later [15].

## 2. Results and Discussion

**Figure 1.**
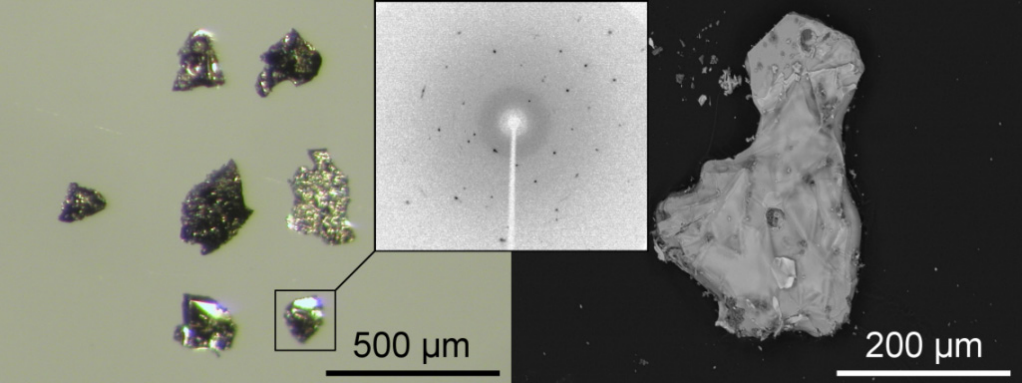
Crystal aggregates of Ca_1−_*_x_*Na*_x_*FFeAs viewed via an optical (**left**) and an electron microscope (**right**). The inset shows a Laue-image of a representative single crystal.

In the present work, we show that it is possible to attain high-temperature superconductivity on alkaline metal-doping of a fluorine containing the 1111 phase for the first time. CaFFeAs [7] was doped with sodium, resulting in the solid-solution series, Ca_1−_*_x_*Na*_x_*FFeAs (*x* = 0 – 0.14), prepared via a solid-state reaction method. Ca_1−_*_x_*Na*_x_*FFeAs consists of small platelet-like crystal aggregates (Figure 1) sufficient in size and quality for single crystal X-ray analysis (about 0.1 × 0.1 mm^2^). Larger single crystals with a composition of Ca_0.89_Na_0.11_FFeAs and a size up to 2 × 2 mm^2^ were grown in a flux of sodium chloride for physical measurements.

### 2.1. Crystal Structure and Crystallographic Data of Ca_1−x_Na_x_FFeAs

**Figure 2.**
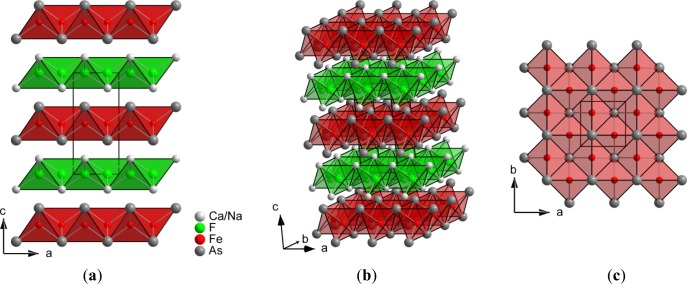
Crystal structure of Ca_1−_*_x_*Na*_x_*FFeAs. (**a**) The view along [010]; (**b**) a three-dimensional picture and (**c**) top view of a [FeAs_4/4_]^−^ layer along [001].

The 1111 phase CaFFeAs [7] crystallizes in the tetragonal ZrCuSiAs-type structure, representing a filled PbFCl variant, in the space group *P*4/*nmm* with *Z* = 2. The structure consists of alternating isotypic cationic [FCa_4/4_]^+^ and anionic [FeAs_4/4_]^−^ layers of edge-connected tetrahedra (Figure 2).

**Figure 3.**
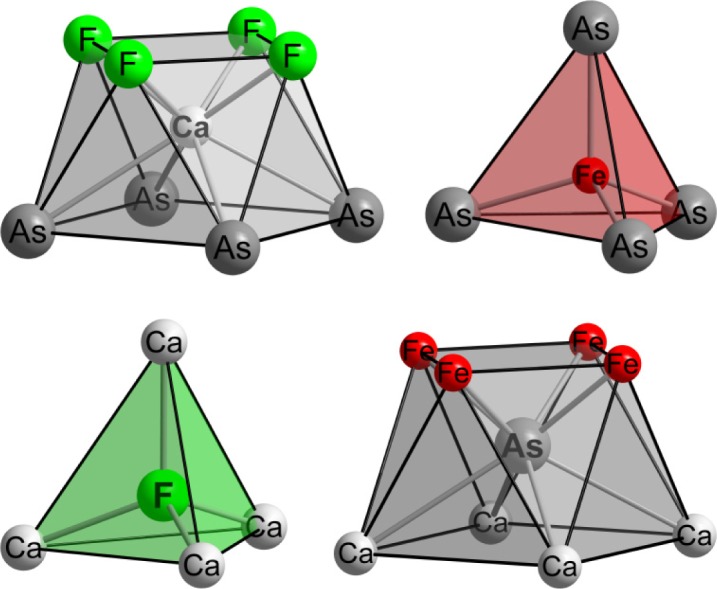
Coordination polyhedra around the four crystallographically independent atoms in Ca_1−_*_x_*Na*_x_*FFeAs.

Sodium partly substitutes calcium in Ca_1−_*_x_*Na*_x_*FFeAs on the 2*c* position. From the four crystallographically independent atoms, iron and fluorine are both tetrahedrally coordinated, whereas arsenic and calcium are surrounded in the form of square antiprisms built from four counter ions of each kind (Figure 3).

Crystallographic data for selected representatives of the substitution series Ca_1−_*_x_*Na*_x_*FFeAs are listed in Table 1. More detailed crystallographic information can be found in the supplementary part (Tables S1–S4).

### 2.2. Phase Analysis of Ca_1−x_Na_x_FFeAs and Discussion

**Figure 4.**
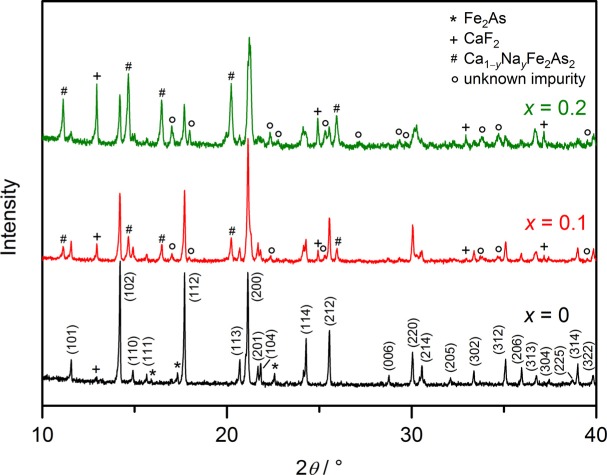
X-ray powder diffraction patterns (Mo-*Kα*_1_ radiation) of the doping series with the target composition Ca_1−_*_x_*Na*_x_*FFeAs (*x* = 0 – 0.2).

Powder X-ray diffractometry verifies the presence of the target compound in each sample of the series. For *x* ≥ 0.1, however, an increasingly larger portion of secondary phases arises (Figure 4).

**Figure 5.**
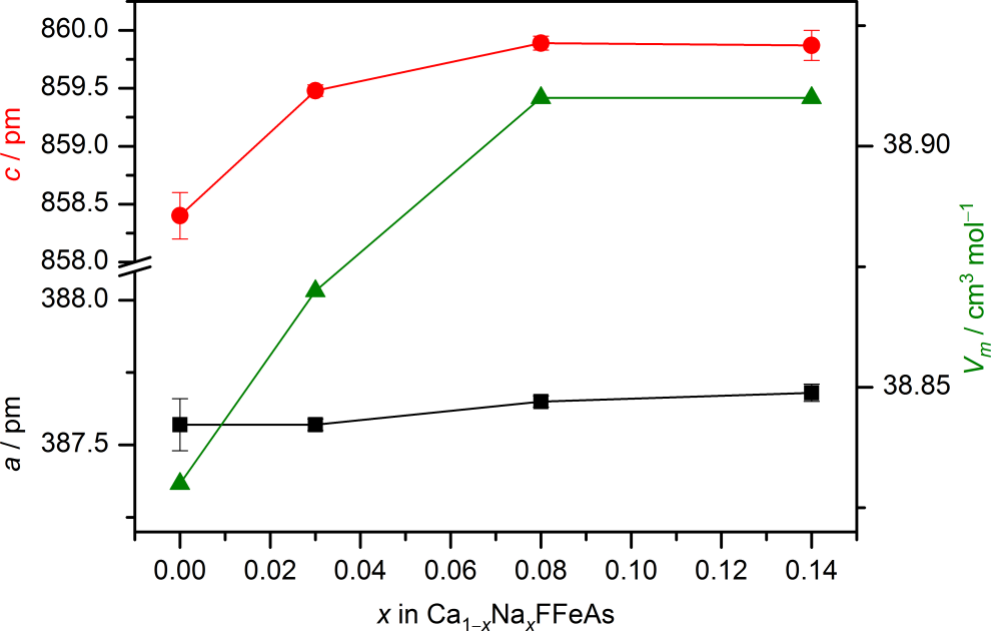
Lattice parameters and molar volumes of Ca_1−_*_x_*Na*_x_*FFeAs phases *versus* the sodium doping grade *x* (from energy dispersive X-ray spectroscopy (EDX)).

Energy dispersive X-ray spectroscopy (EDX) measurements confirm a certain amount of dopants in the target compounds. The crystals of Ca_1−_*_x_*Na*_x_*FFeAs exhibit compositions in a rather narrow range from Ca_0.99_Na_0.01_FFeAs to Ca_0.86_Na_0.14_FFeAs, depending on the sodium concentration used in the synthesis. Single crystals of the series Ca_1−_*_x_*Na*_x_*FFeAs can be structurally refined with the compositions from the EDX analyses (Table 1). Figure 5 shows the development of the lattice parameters, *a* and *c*, as well as the molar volume, *V_m_*, of Ca_1−_*_x_*Na*_x_*FFeAs for *x* = 0, 0.03, 0.08 and 0.14 as a function of the sodium content. The ionic radii with a coordination number of eight are 112 and 118 pm for Ca^2+^ and Na^+^, respectively [19]. The expected increase of the lattice parameters and volume for a rising sodium content is only slightly realized for the *a*-axes, while the increase in the *c*-axes and, subsequently, the volumes are more visible. For the compound Ca_0.86_Na_0.14_FFeAs with the highest degree of substitution, the *a*-axis increases by 0.02% and the *c*-axis by 0.17%, as compared to the unsubstituted compound. An almost unchanged *a*-axis has been observed previously in the case of hole-doped La_0.87_Sr_0.13_OFeAs [15], whereas the unit-cell size even decreases with increasing sodium content in the case of Ca_1−_*_x_*Na*_x_*Fe_2_As_2_ [20]. The observed increase of the *c*-axis for Ca_1−_*_x_*Na*_x_*FFeAs lies in the similar range as in La_0.87_Sr_0.13_OFeAs (0.34%). The rather small rise of the *c*-axis length, as well as the molar volume may indicate a slight overestimation of the sodium content, particularly for the crystal with highest degree of substitution (*x* = 0.14).

A closer look at the equivalent isotropic displacement parameters (see the Supplementary Information, Table S2) shows that the ones for the Ca/Na position remain nearly constant for *x* > 0. The parameters of the other atoms increase with rising sodium content. This effect could be explained with an incremental static displacement of the F, Fe and As atoms for an increasing Na level via the statistical occupation of the 2*c* position (Ca/Na). The atomic coordinates of the Ca_1−_*_x_*Na*_x_*FFeAs crystals do not indicate a significant change with rising substitution grade. This is the reason for the virtually unmodified distances, e.g., *d*(Ca/Na–F) = 233 – 234 pm, *d*(Ca/Na–As) = 316 – 317 pm, *d*(Fe–As) = 240 pm and *d*(Fe–Fe) = 274 pm, and angles, e.g., ∢(As–Fe–As′) = 107.7° – 108.8° and 110.3° – 103.4°, within the series (see Table S4 of the Supplementary Information for details). Therefore, it seems that the variation of the electron count of iron via doping is exclusively responsible for the appearance of superconductivity in this system.

### 2.3. Magnetization and Resistivity Measurements on Ca_1−x_Na_x_FFeAs

**Figure 6.**
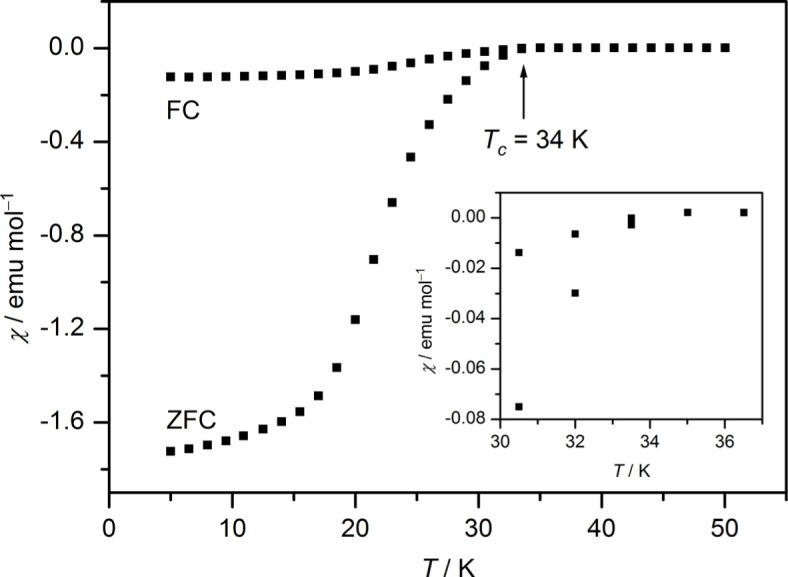
Magnetic susceptibility of polycrystalline Ca_0.9_Na_0.1_FFeAs-containing powder samples in a magnetic field of 100 Oe with a close-up of the transition region.

Magnetization measurements on Ca_1−_*_x_*Na*_x_*FFeAs-containing powder samples indicate the occurrence of superconductivity. In the range of nominal composition of *x* = 0.1 – 0.2, a transition temperature to superconductivity of 34 K was observed, regardless of the doping level (Figure 6).

The superconducting volume fraction in the samples with *x* = 0.1 and 0.2 can be given by about 10% and 24%, respectively. However, highly substituted Ca_1−_*_x_*Na*_x_*Fe_2_As_2_ detected as a side phase in all samples was previously reported to exhibit superconductivity with a nearly identical *T_c_* up to 34 K [20]. For this reason, the superconducting transition observed in the powder samples cannot be ascribed definitively to the target phase. However, it seems that it is at least contributing to the content of the superconducting material, because of the observation that in samples with a higher sodium amount of *x* = 0.3, where an excess of the side phases according to Figure 4 is present and the target phase becomes the minority component, the superconducting volume fraction decreases considerably. Already known hole-doped oxide compounds of the 1111 class show lower transition temperatures (for example, La_0.87_Sr_0.13_OFeAs: *T_c_* = 25.6 K [15] and Pr_0.75_Sr_0.25_OFeAs: *T_c_* = 16.3 K [21]).

**Figure 7.**
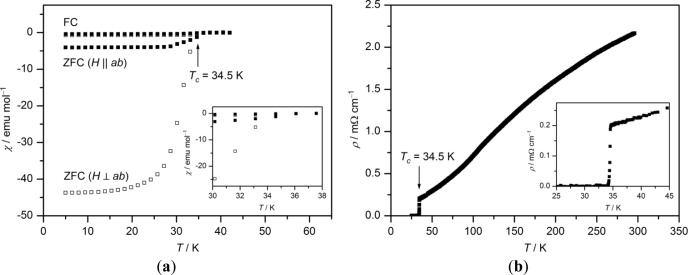
Temperature dependence of the zero-field cooled (ZFC) and field cooled (FC) DC magnetic susceptibility (*μ*_0_*H* = 100 Oe) (**a**) and DC resistivity (**b**) of an oriented single crystal of Ca_0.89_Na_0.11_FFeAs. (The insets are showing close-ups of the transition regions.)

To investigate the characteristics of phase pure sodium-doped 1111, phase resistivity, as well as directional physical measurements of the magnetic susceptibility have been performed on a larger single crystal of Ca_0.89_Na_0.11_FFeAs grown in NaCl flux (Figure 7). Single crystal XRD results in a sodium doping grade of 11(3)% and the following parameters: tetragonal, *P*4/*nmm*, *Z* = 2, *a* = 387.8(1) pm, *c* = 859.9(2) pm, *R*_1_ = 0.034, *wR*_2_ = 0.079, *GooF* = 1.027. The critical temperature apparent at about 34.5 K in both measurements shows that Na-doped CaFFeAs with a sodium content of 11% incidentally exhibits almost the same *T_c_* as Ca_1−_*_x_*Na*_x_*Fe_2_As_2_ (*x* ≈ 0.66) [20].

**Figure 8.**
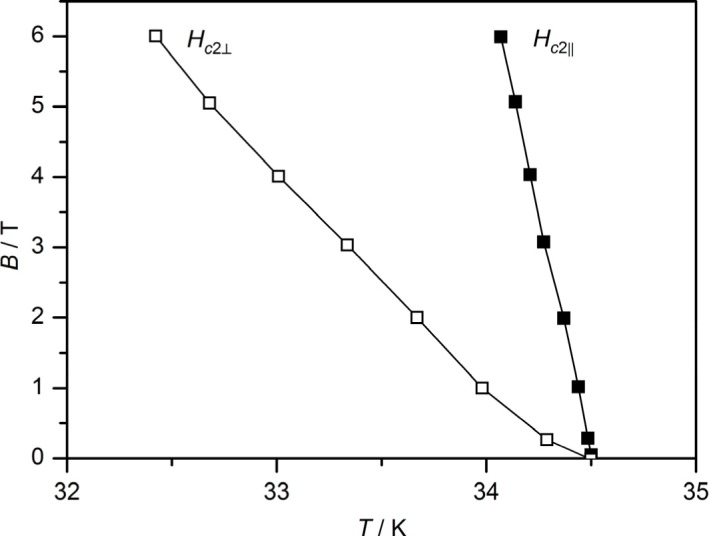
*H*–*T* phase diagram of Ca_0.89_Na_0.11_FFeAs single crystals. (*H_c_*_2_**_⊥_** and *H_c_*_2_**_||_**: upper critical fields perpendicular and parallel to the *ab* plane, respectively.)

The anisotropic properties of Ca_0.89_Na_0.11_FFeAs are available from the temperature dependence of the upper critical fields, which is shown in Figure 8. The anisotropy factor *γ* = *H_c_*_2_**_||_**(0)/*H_c_*_2_**_⊥_**(0) can be calculated from the estimated orbital critical fields, *H_c_*_2_**_⊥_**(0) and *H_c_*_2_**_||_**(0), at 0 K, derived from the Werthamer-Helfand-Hohenberg formula [22], which uses the slope of the *H_c_*_2_ curves at *T_c_*. In the case of Ca_0.89_Na_0.11_FFeAs single crystals, this leads to *H_c_*_2_**_⊥_**(0) = 75.6 T and *H_c_*_2_**_||_**(0) = 370.7 T, which results in *γ* ≈ 5. The received value for the anisotropy factor is in accordance with the literature data for 1111 compounds [23,24]. In contrast 122 phases exhibit smaller values of about two [25,26]. More details on the superconducting properties of Ca_1−_*_x_*Na*_x_*FFeAs (*x* = 0.11) will be discussed in a forthcoming publication [27].

## 3. Experimental Section

The approaches to synthesize Ca_1−_*_x_*Na*_x_*FFeAs were performed via classical solid-state reactions at 1000 °C for 24 h, according to the following equation (*x* = 0 – 0.2).

$$(1-x)\;\mathrm{Ca}F_{\text{2}}+2x\;\mathrm{Na}F+(1-x)\;\mathrm{Ca}\mathrm{As}+2\mathrm{Fe}+(1+x)\;\mathrm{As}\to {\text{ 2 Ca}}_{\text{1}-x}{\mathrm{Na}}_{x}F\mathrm{Fe}\mathrm{As}$$ (1)

The precursors were ground to fine powders and pressed to pellets prior to reaction. Previously, CaAs was synthesized by the reaction of calcium pieces with arsenic powder at 900 °C for 10 h in evacuated silica ampoules. All preparation procedures were carried out in an argon-filled glove box (O_2_, H_2_O < 1 ppm). Small black crystals with a disc-like shape of the target compound were obtained. Single crystals with a size of up to 2 × 2 mm^2^ were available via flux growth in sodium chloride. For this purpose, the educts (CaF_2_, NaF, CaAs, Fe and As), according to Equation (1), are heated with NaCl in an Al_2_O_3_ crucible at 950 °C for 6 h, held at an intermediate temperature of 850 °C for the same duration and, subsequently, cooled to ambient temperature in a period of 24 h (cooling rate: 34.6 °C·h^−1^). These crystals could be separated from the flux manually.

X-ray diffractometry has been performed with the use of Mo-*Kα* radiation (*λ* = 71.07 pm) on a Nonius KappaCCD (Bruker AXS, Karlsruhe, Germany) and a STOE STADI P (Stoe & Cie, Darmstadt, Germany) with a DECTRIS MYTHEN 1K detector for the single-crystal and the powder X-ray measurements, respectively. The single crystals were sealed in glass capillaries, and the powder samples were measured in transmission geometry as thin films on an amorphous adhesive foil. Energy dispersive X-ray spectroscopy (EDX) was carried out via a Thermo Scientific NORAN System 7 X-ray Microanalysis System (Thermo Fisher Scientific, Waltham, MA, USA) with a UltraDry detector. DC-magnetization measurements of oriented single crystals were performed using a Quantum Design MPMS SQUID magnetometer (Quantum Design, San Diego, CA, USA) in the temperature range 5–50 K. *H_c_*_2_ values were determined from DC magnetization in different magnetic fields for field-cooled (FC) and zero-field-cooled (ZFC) measurements in the reversible regime. The electrical resistivity was measured by a standard four-probe DC-method in the temperature range 5 K < *T* < 300 K.

## 4. Conclusions

The results presented here show that it is possible to substitute the fluorine-containing 1111 class of iron pnictides at the alkaline-earth metal position with sodium and to obtain Ca_1−_*_x_*Na*_x_*FFeAs phases. Crystals of Ca_0.89_Na_0.11_FFeAs exhibit superconductivity at 34.5 K, which is intermediate between the observed temperatures for the electron-doped compounds Ca_1−_*_x_*Co*_x_*FFeAs [7] with *T_c_* = 22 K for *x* = 0.1 and Ca_1−_*_x_*Nd*_x_*FFeAs [16] with *T_c_* = 56 K for *x* = 0.6. An estimation of the orbital critical fields, *H_c_*_2_**_⊥_**(0) and *H_c_*_2_**_||_**(0), for Ca_0.89_Na_0.11_FFeAs single crystals results in values of 75.6 and 370.7 T, respectively, which yields an anisotropy ratio of *γ* ≈ 5. This outcome could lead to the discovery of further compounds that are substituted with less valence electron containing elements, which have been very rare, so far. Promising candidates for this purpose could be SrFFeAs and EuFFeAs, with possible substitutes, like sodium and potassium, from an ionic radii point of view. It seems that the variation of the electron count of iron via doping is exclusively responsible for the appearance of superconductivity within this system, because there is no significant change in the interatomic distances and angles with increasing sodium content.

## Figures and Tables

**Table 1.** Crystallographic data for selected crystals of Ca_1−_*_x_*Na*_x_*FFeAs.

*x* in Ca_1−_*_x_*Na*_x_*FFeAs		0	0.03	0.08	0.14
Crystal system		tetragonal			
Space group		*P*4/*nmm* (*Z* = 2)			
Lattice parameters	*a*/pm *c*/pm *c*/*a*	387.57(9) 858.4(2) 2.215	387.57(2) 859.48(5) 2.218	387.65(2) 859.89(6) 2.218	387.68(3) 859.87(9) 2.218
*V_m_*/cm^3^·mol^−1^		38.83	38.87	38.91	38.91
*D_x_*/g·cm^−3^		4.89	4.87	4.84	4.82
Diffractometer		Nonius KappaCCD (Bruker AXS, Karlsruhe, Germany)			
Radiation		Mo-*Kα* (*λ* = 71.07 pm)			
Collected reflections		1589	2278	3232	1622
Unique reflections		118	145	211	118
*R_int_*, *R_σ_*		0.083, 0.028	0.060, 0.018	0.068, 0.021	0.104, 0.039
*R*_1_, *wR*_2_		0.076, 0.157	0.018, 0.044	0.022, 0.053	0.026, 0.058
*GooF*		1.178	1.168	1.166	1.165
Solution and refinement		Program package *SHELX-97* [17,18]			

## Supplementary Materials

Supplementary File 1

Supplementary File 2
