# Supplementary material for: Synthesis and Characterization of Superconducting Ca1−xNaxFFeAs
Source: Materials (Basel). 2014 Mar 7;7(3):1984–94. doi: 10.3390/ma7031984 (PMC5453247; doi:10.3390/ma7031984)
Supplement: Supplementary File 1 [file materials-07-01984-s001.pdf]

Supplementary Information

Table S1. Crystallographic data for selected crystals of the series $Ca_{1-x}Na_xFFeAs$. (Na contents are adopted from EDX analyses.)

***x* in $Ca_{1-x}Na_xFFeAs$**		**0**	**0.03**	**0.08**	**0.14**
Crystal system		tetragonal			
Space group		*P*4/*nmm* (No. 129)			
Lattice parameters	*a*/pm	387.57(9)	387.57(2)	387.65(2)	387.68(3)
	c/pm	858.4(2)	859.48(5)	859.89(6)	859.87(9)
	c/*a*	2.215	2.218	2.218	2.218
Formula units per unit cell *Z*		2			
Molar volume $V_m/cm^3 \cdot mol^{-1}$		38.83	38.87	38.91	38.91
Calculated density $D_x/g \cdot cm^{-3}$		4.89	4.87	4.84	4.82
Diffractometer		Nonius KappaCCD (Bruker AXS, Karlsruhe, Germany)			
Radiation		Mo-$K\alpha$ (λ = 71.07 pm)			
hkl range $\pm h_{max}$, $\pm k_{max}$, $\pm l_{max}$		5, 5, 11	5, 5, 12	6, 6, 13	5, 5, 11
$2\theta_{max}/°$		58.80	60.66	71.20	56.27
F(000)		176	176	176	176
Absorption correction		numerical (Program *HABITUS* [1])			
Absorption coefficient μ/mm^{-1}		20.24	20.22	20.20	20.20
Extinction coefficient *g*		–	0.0239	0.0226	–
Collected reflections		1589	2278	3232	1622
Unique reflections		118	145	211	118
Reflexions with $\|F_o\| \geq 4\sigma(F_o)$		99	142	202	109
Refined parameters		11	12	12	11
R_{int}, R_σ		0.083, 0.028	0.060, 0.018	0.068, 0.021	0.104, 0.039
Solution and refinement		Program package *SHELX-97* [2,3]			
Scattering factors		*International Tables, Vol. C* [4]			
R_1, R_1 with $\|F_o\| \geq 4\sigma(F_o)$		0.076, 0.062	0.018, 0.018	0.022, 0.021	0.026, 0.024
wR_2, *GooF*		0.157, 1.178	0.044, 1.168	0.053, 1.166	0.058, 1.165
Residual electron density ρ_{max}, $\rho_{min}/10^{-6} \cdot pm^{-3}$		2.03, −1.73	0.59, −0.68	1.30, −0.87	0.97, −0.49

Table S2. Atomic coordinates and equivalent isotropic displacement parameters for selected crystals of the series $Ca_{1-x}Na_xFFeAs$.

**Atom**	**Site**	***x*/*a***	***y*/*b***	***z*/*c***	**$U_{eq}^{(a)}/pm^2$**
		CaFFeAs			
Ca	2*c*	1/4	1/4	0.1514(6)	265(13)
F	2*a*	3/4	1/4	0	263(35)
Fe	2*b*	3/4	1/4	1/2	251(11)
As	2*c*	1/4	1/4	0.6645(3)	251(9)

Table S2. *Cont.*

**Atom**	**Site**	***x*/*a***	***y*/*b***	***z*/*c***	**U_{eq}[a]/pm²**
			$Ca_{0.97}Na_{0.03}FFeAs$		
Ca/Na[b]	2*c*	$^{1}/_{4}$	$^{1}/_{4}$	0.15111(12)	84(3)
F	2*a*	$^{3}/_{4}$	$^{1}/_{4}$	0	90(6)
Fe	2*b*	$^{3}/_{4}$	$^{1}/_{4}$	$^{1}/_{2}$	74(2)
As	2*c*	$^{1}/_{4}$	$^{1}/_{4}$	0.66461(5)	75(2)
			$Ca_{0.92}Na_{0.08}FFeAs$		
Ca/Na[c]	2*c*	$^{1}/_{4}$	$^{1}/_{4}$	0.15072(10)	92(2)
F	2*a*	$^{3}/_{4}$	$^{1}/_{4}$	0	115(5)
Fe	2*b*	$^{3}/_{4}$	$^{1}/_{4}$	$^{1}/_{2}$	88(2)
As	2*c*	$^{1}/_{4}$	$^{1}/_{4}$	0.66444(4)	90(2)
			$Ca_{0.86}Na_{0.14}FFeAs$		
Ca/Na[d]	2*c*	$^{1}/_{4}$	$^{1}/_{4}$	0.1513(2)	94(4)
F	2*a*	$^{3}/_{4}$	$^{1}/_{4}$	0	136(11)
Fe	2*b*	$^{3}/_{4}$	$^{1}/_{4}$	$^{1}/_{2}$	105(4)
As	2*c*	$^{1}/_{4}$	$^{1}/_{4}$	0.66440(10)	112(3)

[a] $U_{eq} = {}^{1}/_{3}(U_{11} + U_{22} + U_{33})$; [b] Site occupation (from EDX): 97% Ca, 3% Na; [c] Site occupation (from EDX): 92% Ca, 8% Na; [d] Site occupation (from EDX): 86% Ca, 14% Na.

Table S3. Anisotropic displacement parameters (U_{ij}[a] in pm²) for selected crystals of the series $Ca_{1-x}Na_xFFeAs$. ($U_{12} = U_{13} = U_{23} = 0$ for all atoms.)

**Atom**	**$U_{11} = U_{22}$**	**U_{33}**
	CaFFeAs	
Ca	241(19)	315(24)
F	218(55)	352(67)
Fe	195(14)	365(18)
As	212(11)	329(15)
	$Ca_{0.97}Na_{0.03}FFeAs$	
Ca/Na	73(3)	106(5)
F	74(8)	123(13)
Fe	62(3)	99(4)
As	69(2)	87(3)
	$Ca_{0.92}Na_{0.08}FFeAs$	
Ca/Na	82(2)	114(4)
F	104(7)	137(11)
Fe	77(2)	110(3)
As	85(2)	99(2)
	$Ca_{0.86}Na_{0.14}FFeAs$	
Ca/Na	77(6)	127(9)
F	146(17)	116(24)
Fe	84(5)	148(7)
As	97(4)	141(5)

[a] given in the expression $\exp[-2\pi^2(a^{*2}h^2U_{11} + b^{*2}k^2U_{22} + c^{*2}l^2U_{33} + 2b^*c^*klU_{23} + 2a^*c^*hlU_{13} + 2a^*b^*hkU_{12})]$.

Table S4. Interatomic distances and angles for selected crystals of the series $Ca_{1-x}Na_xFFeAs$.

Distance	*d*/pm	Multiplicity	Distance	*d*/pm	Multiplicity
		CaFFeAs			
Ca–F	233.4(3)	(4×)	Fe–Fe	274.05(7)	(4×)
Ca–As	316.4(3)	(4×)	Fe–As	239.76(16)	(4×)
		$Ca_{0.97}Na_{0.03}FFeAs$			
Ca/Na–F	233.28(6)	(4×)	Fe–Fe	274.05(1)	(4×)
Ca/Na–As	316.53(6)	(4×)	Fe–As	239.94(3)	(4×)
		$Ca_{0.92}Na_{0.08}FFeAs$			
Ca/Na–F	233.16(5)	(4×)	Fe–Fe	274.11(1)	(4×)
Ca/Na–As	316.86(5)	(4×)	Fe–As	239.92(2)	(4×)
		$Ca_{0.86}Na_{0.14}FFeAs$			
Ca/Na–F	233.46(11)	(4×)	Fe–Fe	274.13(3)	(4×)
Ca/Na–As	316.63(11)	(4×)	Fe–As	239.91(6)	(4×)
Angle	∢/ °	Multiplicity	Angle	∢/ °	Multiplicity
		CaFFeAs			
F–Ca–F'	71.92(10)	(4×)	As–Fe–As'	107.85(10)	(2×)
	112.3(2)	(2×)		110.29(5)	(4×)
F–Ca–As	76.68(4)	(8×)	Ca–As–Ca'	75.55(8)	(4×)
	141.90(2)	(8×)		120.06(18)	(2×)
As–Ca–As'	75.55(8)	(4×)	Ca–As–Fe	78.41(7)	(8×)
	120.06(18)	(2×)		142.12(3)	(8×)
Ca–F–Ca'	108.08(10)	(4×)	Fe–As–Fe'	69.71(5)	(4×)
	112.3(2)	(2×)		107.85(10)	(2×)
		$Ca_{0.97}Na_{0.03}FFeAs$			
F–Ca/Na–F'	71.94(2)	(4×)	As–Fe–As'	107.73(2)	(2×)
	112.34(4)	(2×)		110.35(1)	(4×)
F–Ca/Na–As	76.70(1)	(8×)	Ca/Na–As–Ca/Na'	75.50(2)	(4×)
	141.92(1)	(8×)		119.95(4)	(2×)
As–Ca/Na–As'	75.50(2)	(4×)	Ca/Na–As–Fe	78.50(1)	(8×)
	119.95(4)	(2×)		142.14(1)	(8×)
Ca/Na–F–Ca/Na'	108.06(2)	(4×)	Fe–As–Fe'	69.65(1)	(4×)
	112.34(4)	(2×)		107.74(2)	(2×)
		$Ca_{0.92}Na_{0.08}FFeAs$			
F–Ca/Na–F'	72.00(2)	(4×)	As–Fe–As'	107.78(1)	(2×)
	112.46(3)	(2×)		110.32(1)	(4×)
F–Ca/Na–As	76.72(1)	(8×)	Ca/Na–As–Ca/Na'	75.43(1)	(4×)
	141.94(1)	(8×)		119.78(3)	(2×)
As–Ca/Na–As'	75.43(1)	(4×)	Ca/Na–As–Fe	78.55(1)	(8×)
	119.78(3)	(2×)		142.17(1)	(8×)
Ca/Na–F–Ca/Na'	108.00(2)	(4×)	Fe–As–Fe'	69.68(1)	(4×)
	112.46(3)	(2×)		107.78(1)	(2×)

Table S4. *Cont.*

Distance	*d*/pm	Multiplicity	Distance	*d*/pm	Multiplicity
		$Ca_{0.86}Na_{0.14}FFeAs$			
F–Ca/Na–F'	71.90(4)	(4×)	As–Fe–As'	107.80(4)	(2×)
	112.26(8)	(2×)		110.32(2)	(4×)
F–Ca/Na–As	76.74(1)	(8×)	Ca/Na–As–Ca/Na'	75.50(3)	(4×)
	141.93(1)	(8×)		119.94(6)	(2×)
As–Ca/Na–As'	75.50(3)	(4×)	Ca/Na–As–Fe	78.48(3)	(8×)
	119.94(6)	(2×)		142.14(1)	(8×)
Ca/Na–F–Ca/Na'	108.10(4)	(4×)	Fe–As–Fe'	69.68(2)	(4×)
	112.26(8)	(2×)		107.80(4)	(2×)

References

1. Herrendorf, W.; Bärnighausen, H. *HABITUS: Program for the Optimization of the Crystal Shape for the Numerical Absorption Correction*; Universities of Gießen and Karlsruhe: Gießen, Karlsruhe, Germany, 1997.
2. Sheldrick, G.M. *SHELX-97: Program Package for the Determination of Crystal Structures by Single Crystal X-Ray and Neutron Diffraction*; University of Göttingen: Göttingen, Germany, 1997.
3. Sheldrick, G.M. A short history of SHELX. *Acta Crystallogr.* **2008**, *64*, 112–122.
4. Prince, E. *International Tables for Crystallography*, 3rd ed.; Kluwer Academic Publishers: Dordrecht, Netherlands, 2004.
